# Ectopic Mineralization and Conductive Hearing Loss in *Enpp1^asj^* Mutant Mice, a New Model for Otitis Media and Tympanosclerosis

**DOI:** 10.1371/journal.pone.0168159

**Published:** 2016-12-13

**Authors:** Cong Tian, Belinda S. Harris, Kenneth R. Johnson

**Affiliations:** 1 The Jackson Laboratory, Bar Harbor, Maine, United States of America; 2 Graduate School of Biomedical Science and Engineering, University of Maine, Orono, Maine, United States of America; Universite de Nantes, FRANCE

## Abstract

Otitis media (OM), inflammation of the middle ear, is a common cause of hearing loss in children and in patients with many different syndromic diseases. Studies of the human population and mouse models have revealed that OM is a multifactorial disease with many environmental and genetic contributing factors. Here, we report on otitis media-related hearing loss in *asj* (ages with stiffened joints) mutant mice, which bear a point mutation in the *Enpp1* gene. Auditory-evoked brainstem response (ABR) measurements revealed that around 90% of the mutant mice (*Enpp1*^*asj/asj*^) tested had moderate to severe hearing impairment in at least one ear. The ABR thresholds were variable and generally elevated with age. We found otitis media with effusion (OME) in all of the hearing-impaired *Enpp1*^*asj/asj*^ mice by anatomic and histological examinations. The volume and inflammatory cell content of the effusion varied among the *asj* mutant mice, but all mutants exhibited a thickened middle ear epithelium with fibrous polyps and more mucin-secreting goblet cells than controls. Other abnormalities observed in the *Enpp1* mutant mice include over-ossification at the round window ridge, thickened and over-calcified stapedial artery, fusion of malleus and incus, and white patches on the inside of tympanic membrane, some of which are typical symptoms of tympanosclerosis. An excessive yellow discharge was detected in the outer ear canal of older *asj* mutant mice, with 100% penetrance by 5 months of age, and contributes to the progressive nature of the hearing loss. This is the first report of hearing loss and ear pathology associated with an *Enpp1* mutation in mice. The *Enpp1*^*asj*^ mutant mouse provides a new animal model for studying tympanosclerotic otitis and otitis media with effusion, and also provides a specific model for the hearing loss recently reported to be associated with human *ENPP1* mutations causing generalized arterial calcification of infancy and hypophosphatemic rickets.

## Introduction

While many genes have been discovered that underlie Mendelian forms of sensorineural hearing loss (http://hereditaryhearingloss.org/), most cases of conductive hearing loss, often caused by otitis media and tympanosclerosis, have complex and poorly understood etiologies. Otitis media is an inflammation of the middle ear and is among the most common childhood illnesses. Heritability studies have shown that genetic factors can play an important role in otitis media susceptibility, but few contributing genes have been identified in human populations [1]. In contrast to human studies, a growing number of mouse mutations have been identified that manifest a high incidence of otitis media, including *Eya4*, *Tlr4*, *p73*, *MyD88*, *Fas*, *E2f4*, *Plg*, *Fbxo11*, *Evi1* [1,2], *Sh3pxd2b* [3], *Rpl38* [4], *Isl1* [5], *Chd7* [6], *Lmna1* [7], *Phex* [8], *Oxgr1* [9], *Tgif1* [10], and *Mcph1* [11]. The wide diversity of these genes and their mutant pathologies, including craniofacial abnormalities with Eustachian tube malformations and innate immune response defects, underscores the complex nature of otitis media.

Here we report on the conductive hearing loss associated with a recessive ENU-induced missense mutation of the ectonucleotide pyrophosphatase/phosphodiesterase 1 gene (*Enpp1*) that was discovered at The Jackson Laboratory and named "ages with stiffened joints" (*asj*) because of the progressive ankylosis and osteoarthritis exhibited by mutant mice [12]. The *Enpp1* gene encodes an enzyme (ENPP1) that regulates soft-tissue calcification and bone mineralization by producing inorganic pyrophosphate, a major inhibitor of calcification. A previous analysis of *asj* mutant mice found that enzymatic activity of ENPP1 in the liver and inorganic pyrophosphate (PPi) levels in plasma were markedly reduced [13]. Other mouse mutations of *Enpp1* have been reported, including the naturally occurring "tiptoe walking" (*ttw*) nonsense mutation [14], an ENU-induced C397S missense mutation [15], and a genetically engineered knockout mutation [16]. The *Enpp1* mutant mice in these studies exhibited extensive mineralization defects in a number of tissues, including spinal ligaments, long bones, articular cartilage, heart, aorta, arterial blood vessels, vibrissae, liver, kidneys, and retina. The effects of the *Enpp1* mutations on auditory function and middle and inner ear histology of mice, however, were not examined.

We show here that *Enpp1*^*asj*^ mutant mice exhibit a conductive hearing loss that is associated with middle and inner ear mineralization abnormalities. These mice provide a new animal model for studies of otitis media and tympanosclerosis and for the hearing loss recently shown to be associated with human *ENPP1* mutations causing generalized arterial calcification of infancy and hypophosphatemic rickets [17,18].

## Materials and Methods

### Mice

The recessive *asj* mutation was discovered at The Jackson Laboratory in 2004 in the C57BL/6J progeny of an ENU treated C57BL/6J male. The mutant strain name is C57BL/6J-*Enpp1*^*asj*^/GrsrJ and is available from The Jackson Laboratory (Stock #012810). Experimental mice were housed in the Research Animal Facility of The Jackson Laboratory, and all procedures involving their use were approved by the Institutional Animal Care and Use Committee. The Jackson Laboratory is accredited by the American Association for the Accreditation of Laboratory Animal Care.

### Genotyping the *Enpp1*^*asj*^ mutation

The *asj* mutation is a single nucleotide substitution of T to A in exon 7 of the *Enpp1* gene resulting in an amino acid substitution from valine to aspartic acid at residue 246 (p.V246D) of protein Reference Sequence NP_032839. The T to A point mutation (nt 848 of mRNA Reference Sequence NM_008813) creates a new *TaqI* restriction site (TCGA), which is the basis of the genotyping method described by Harris and colleagues [12] and that was used in this study to distinguish wild-type from mutant alleles.

### Assessment of hearing by ABR

Hearing was evaluated in anesthetized (Avertin, 0.4 mg/g mouse mass) wild-type, *Enpp1*^*asj/+*^, and *Enpp1*^*asj/asj*^ mice by measuring auditory brainstem response (ABR). A computer-aided evoked potential system (Intelligent Hearing Systems) was used to measure mouse ABR thresholds as previously described [19].

### Histological analyses of middle and inner ears

Histological analyses of the middle and inner ears were performed following the methods described previously [20]. Briefly, middle and inner ears from *Enpp1*^*asj/asj*^ mice and wild-type mice were dissected after transcardial perfusion with Bouin’s fixative. Ear samples were immersed in Bouin’s fixative (7 days for one-month-old mice and 30 days for mice older than 6 months) and embedded in paraffin. Sections (7 mm) were cut and mounted onto Fisher Superfrost Plus slides (Fisher Scientific, Pittsburgh, PA) and counterstained in hematoxylin/eosin (H&E). Goblet cells, whose sole function is to secrete mucus, were identified by Mayer’s Mucicarmine staining method following the protocol provided by Electron Microscopy Sciences (Catalog #26320). The stepedial artery was dissected from 6-month-old control and mutant mice, sectioned without decalcification, and stained with Alizarin red following standard procedures [21].

### Evaluation of pathology of middle ears

A scoring system of -/+/++/+++ was used to assess the severity of pathology in the middle ears following a previously described method with modifications [22]. Histological analysis of the middle ears of control and mutant mice was performed using an Olympus BX51 microscope. A ‘-’ symbol was assigned when the pathology is absent in the middle ear. A ‘+’ symbol was assigned when pathology was very scarce in the middle ear. A ‘++’ symbol was assigned when pathology was more prevalent, but not to the point of spanning the entire middle ear. A ‘+++’ symbol was assigned when pathology spanned the entire middle ear. The following pathologies were evaluated by this scoring system: middle ear effusion, inflammatory cell infiltration, tissue proliferation (epithelial hyperplasia), abnormal tissue at the Eustachian tube opening in the middle ear cavity, ectopic mineralization, and clusters of goblet cells.

### Scanning electron microscopy (SEM)

Middle ears from *Enpp1*^*asj/asj*^ and wild-type mice were dissected after transcardial perfusion with 4% paraformaldehyde (PFA) and then immersed in 2.5% glutaraldehyde in 0.1 M cacodylate buffer (pH = 7.2) at 4°C overnight. Dissection was performed to expose the middle ear cavities. After three 15-minute washes with cacodylate buffer (pH = 7.2) at room temperature, samples were post-fixed with the osmium-thiocarbohydrazide-osmium-thiocarbohydrazide-osmium (OTOTO) method [23], and dehydrated in increasing concentrations of ethanol at 4°C. Samples were critical point dried with Hexamethyldisilazane (HMDS) and then air dried in fume hood. Samples were then coated with 15nm gold and analyzed in a Hitachi S-3000 scanning electron microscope (Hitachi, Tokyo, Japan) at 20 kV.

## Results

### Hearing impairment in *Enpp1*^*asj*^ mutant mice

**Fig 1:**
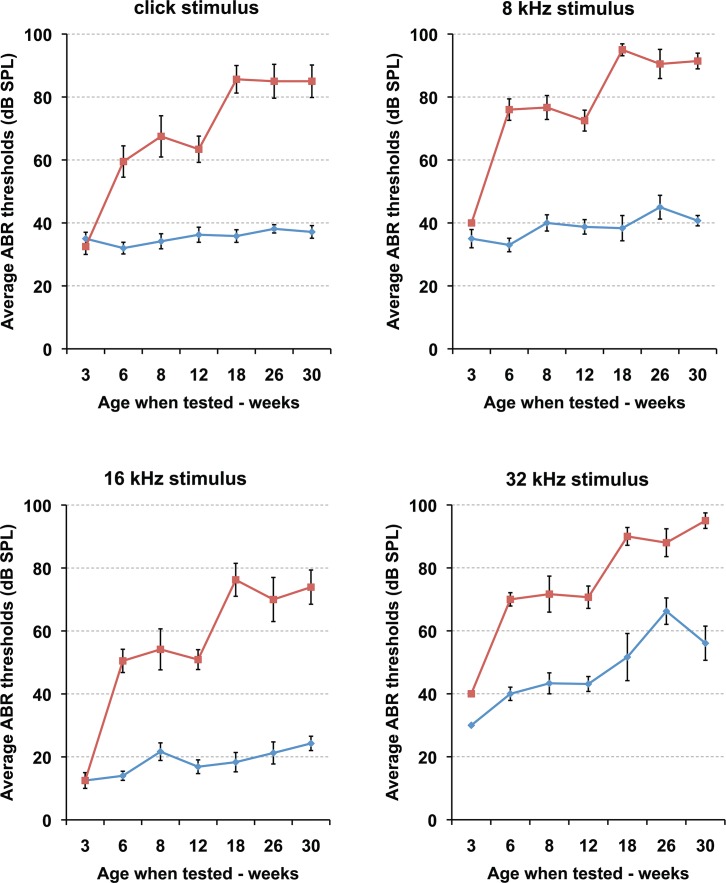
Progressive hearing loss in *Enpp1*^*asj/asj*^ mice. ABR threshold means are shown for *Enpp1*^*asj/asj*^ mice and littermate control mice, tested at the ages of 3 (n = 6 mutant ears/8 control ears), 6 (n = 10/10), 8 (n = 6/6), 12 (n = 22/16), 18 (n = 8/6), 26 (n = 10/8), and 30 (n = 12/14) weeks. Starting from 6 weeks of age, the mutant mice exhibit significantly higher mean ABR threshold values at all the stimulus frequencies tested (click, 8 kHz, 16 kHz, 32 kHz) compared to those of the littermate controls. Thresholds of mutant mice continue to increase with age and by 18 weeks most of the *Enpp1*^*asj/asj*^ mice are profoundly hearing impaired. The increase in 32 kHz ABR thresholds of the control mice is due to the B6 background, a strain known to exhibit age-related hearing loss starting at high frequencies. Error bars indicate standard errors of the mean.

Hearing in the *Enpp1* mutant mice and age-matched controls was evaluated by ABR threshold analysis. Initial ABR measurements of three-month-old mutant mice revealed a moderate to severe hearing loss with threshold increases of 25–35 decibels (dB) compared to the normal thresholds in the control mice for both click and all pure tone stimuli (8 kHz, 16 kHz, 32 kHz). To further characterize the hearing ability of *Enpp1*^*asj/asj*^ mice and to determine whether the hearing impairment is progressive with age, recurrent ABR measurements were performed in *Enpp1*^*asj/asj*^, *Enpp1*^*asj/+*^, and *Enpp1*^*+/+*^ littermates from 3 weeks to 30 weeks of age (Fig 1, Table 1, Table 2). *Enpp1*^*asj/+*^ and *Enpp1*^*+/+*^ mice (grouped as controls) showed normal ABR thresholds even at 30 weeks of age, whereas *Enpp1*^*asj/asj*^ mice exhibited elevated thresholds by 6 weeks of age. Hearing impairment is not congenital as mutant mice had normal ABR thresholds at weaning age (3 weeks). Hearing loss in mutant mice progressed in two stages: average ABR thresholds increased by about 30–35 dB between 3 and 6 weeks of age, were relatively stable between 6 and 12 weeks, increased another 20–25 db between 12 and 18 weeks, and remained stable between 18 and 30 weeks. At 12 weeks of age, only one out of 11 *Enpp1*^*asj/asj*^ mice tested showed normal thresholds in both ears (Table 1), giving a rough estimate of hearing loss penetrance of about 90%. No head bobbing or circling behaviors were observed in the mutant mice at all ages, indicating normal vestibular function.

**Table 1:** ABR thresholds of *asj/asj* mutant mice

Mouse	Age	Test Age		Right Ear thresholds (db SPL)				Left Ear thresholds (dB SPL)			
ID *	Group	Days	Sex	Click	8 kHz	16 kHz	32 kHz	Click	8 kHz	16 kHz	32 kHz
5729	3 wk	23	F	30	30	10	35	40	45	15	35
5055 *	3 wk	22	M	30	40	10	40	35	40	15	40
5738	3 wk	25	M	30	20	10	40	30	30	10	35
4195 *	6 wk	38	M	35	70	50	80	75	80	60	70
5001	6 wk	39	F	70	80	40	70	45	50	30	60
5003	6 wk	39	M	60	80	40	80	60	90	50	70
4094 *	6 wk	41	F	70	80	70	70	75	80	55	70
4098	6 wk	41	M	35	70	50	60	70	80	60	70
3915	8 wk	50	F	75	80	50	85	70	75	40	80
3891 *	8 wk	55	F	35	60	35	45	75	75	55	75
3893 *	8 wk	55	M	75	85	75	70	75	85	70	75
3902	12 wk	75	F	45	50	30	40	40	50	30	40
3904	12 wk	75	M	65	75	40	70	40	40	40	50
3905 *	12 wk	75	M	30	50	20	40	80	85	60	90
3899 *	12 wk	75	F	60	65	50	70	70	85	50	65
4038	12 wk	89	F	100	90	60	90	80	80	50	70
4039	12 wk	89	F	65	80	40	70	80	80	50	80
4040	12 wk	89	M	70	80	60	90	70	85	50	90
4030	12 wk	101	F	90	90	80	90	90	90	80	90
4033	12 wk	101	M	60	80	70	70	40	60	60	70
4034	12 wk	101	M	40	70	50	70	70	80	50	80
4035	12 wk	101	M	70	80	50	70	40	50	50	60
5005	18 wk	123	F	95	100	95	100	100	100	95	100
5007	18 wk	125	F	90	100	70	90	60	90	50	90
5010	18 wk	136	F	90	100	70	85	80	90	80	75
5008	18 wk	144	F	80	90	70	90	90	90	80	90
3884 *	26 wk	172	M	100	100	90	90	75	100	60	100
3885 *	26 wk	172	F	90	100	70	80	100	100	100	100
3886	26 wk	172	M	100	100	90	90	70	85	70	60
3906	26 wk	188	F	100	100	80	100	95	90	70	100
3913	26 wk	188	M	60	70	40	90	60	60	30	70
3106	30 wk	202	F	100	100	100	100	90	90	70	100
3107	30 wk	202	F	100	100	90	100	100	100	90	100
3283	30 wk	204	F	100	100	90	100	100	100	90	100
3098	30 wk	206	F	80	90	80	90	50	80	40	70
3102	30 wk	206	M	70	80	70	90	90	90	60	90
3286	30 wk	223	M	80	90	50	100	50	80	50	100
3290	30 wk	223	M	100	100	90	100	80	80	65	90

* Highlighted ID numbers indicate ears were examined for pathology

**Table 2:** ABR thresholds of +/+ or +/*asj* control mice

Mouse	Age	Test Age		Right Ear thresholds (db SPL)				Left Ear thresholds (dB SPL)			
ID *	Group	Days	Sex	Click	8 kHz	16 kHz	32 kHz	Click	8 kHz	16 kHz	32 kHz
5044	3 wk	21	F	35	30	20	40	40	40	20	40
5045	3 wk	21	M	35	30	20	35	30	30	20	30
5054	3 wk	22	F	35	40	10	30	30	30	10	30
5056	3 wk	22	M	33	30	10	30	40	40	20	30
4193 *	6 wk	38	M	30	30	10	40	35	30	20	40
5002	6 wk	39	F	20	20	10	30	40	30	15	40
5004	6 wk	39	M	30	40	10	40	30	40	10	40
4095 *	6 wk	41	F	30	40	20	50	35	40	20	50
4097	6 wk	41	M	30	30	10	40	40	30	15	30
3914	8 wk	50	F	30	40	25	50	35	40	30	50
3888	8 wk	55	F	25	30	20	40	35	40	20	40
3892 *	8 wk	55	M	40	50	25	50	40	40	10	30
3900 *	12 wk	75	F	30	35	10	40	30	35	20	40
3901	12 wk	75	F	30	40	25	50	60	60	35	60
3903	12 wk	75	F	25	30	20	40	35	30	20	40
4036	12 wk	89	F	35	30	10	30	50	35	15	40
4037	12 wk	89	F	50	55	35	70	35	40	20	40
4032	12 wk	101	M	25	30	10	40	35	30	10	40
4029	12 wk	101	F	35	40	10	40	40	40	10	40
4031	12 wk	101	M	30	40	10	40	35	50	10	40
5006	18 wk	123	F	35	30	10	30	35	40	20	40
5011	18 wk	136	F	30	50	10	60	35	50	20	40
5009	18 wk	144	F	35	30	30	60	45	30	20	80
3883 *	26 wk	172	F	40	40	20	80	40	50	20	60
3887 *	26 wk	172	M	40	60	30	70	40	60	40	50
3910	26 wk	188	M	35	30	10	70	40	40	10	80
3911	26 wk	188	F	30	40	20	50	40	40	20	70
3110	30 wk	202	F	30	40	20	40	30	40	20	40
3112	30 wk	202	M	30	40	20	50	30	40	20	50
3286	30 wk	204	M	30	30	20	45	40	40	20	50
3101	30 wk	206	M	35	40	20	40	45	40	30	40
3104	30 wk	206	M	30	30	10	40	40	40	20	45
5014	30 wk	231	F	40	50	30	80	50	40	30	90
5012	30 wk	234	F	40	50	40	85	50	50	40	90

* Highlighted ID numbers indicate ears were examined for pathology

### Otitis media with effusion in *Enpp1*^*asj/asj*^ mice

To assess the causes of hearing impairment in the *Enpp1*^*asj/asj*^ mice, anatomical analysis of the middle and inner ears was performed after the completion of ABR measurements. We processed the whole bullae for histological studies, which allowed us to observe both middle and inner ear morphology. We did not observe any obvious inner ear defects in the *asj* mutant mice, such as hair cell and spiral ganglion cell loss, or stria vascularis degeneration. However, we observed major histopathology in the middle and outer ear, with details described below.

#### a. Middle ear effusion and epithelial proliferation in *Enpp1* mutant mice

**Fig 2:**
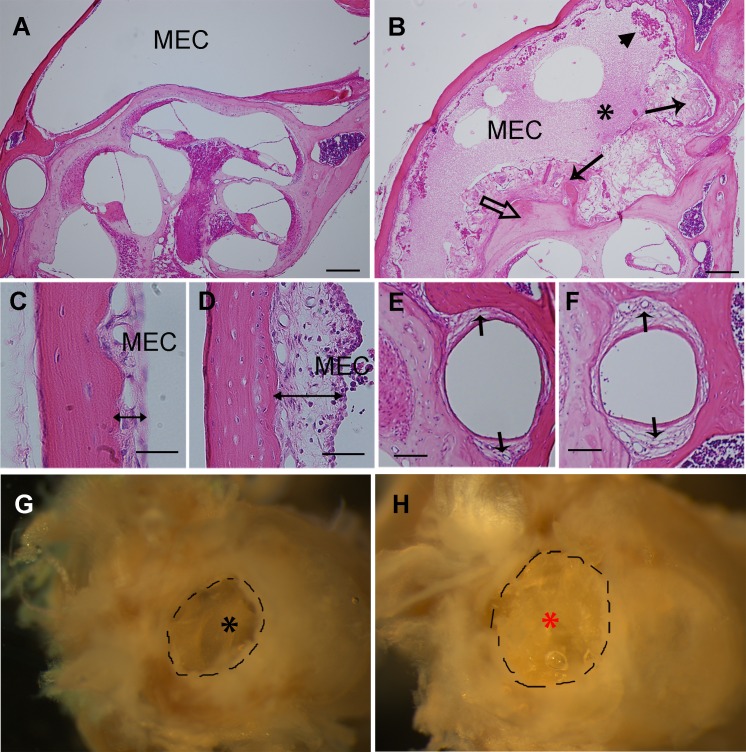
Otitis media in *Enpp1*^*asj/asj*^ mice. A, B: Representative images of pathological changes in the middle ears of *Enpp1*^*asj*^ mutant mice (B) compared with controls (A) at the age of 5 months. The middle ear cavity (MEC) of *Enpp1*^*asj*^ mutant mice is filled with effusions (black asterisk), fibroblastic and amorphous tissue masses (long arrows), and inflammatory cells (arrow head). Ectopic mineralization of the otic capsule is also evident in mutant mice (open arrow in B). Control mice show a clear middle ear cavity without fibroblastic proliferation (A). C, D: The thickness of the middle ear epithelium (double headed arrows) is greater in *Enpp1*^*asj*^ mutant mice (D) than controls (C). E, F: The stapedial artery wall (arrows) is thicker in *Enpp1*^*asj*^ mutant mice (F) than controls (E). G, H: Representative images of discharge in mutant mice (H) and littermate controls (G) at the age of 5 months. The external ear cavity of the mutant mice is filled with discharge (red asterisk), while control mice have a clear external ear canal and easily observable tympanic membrane (black asterisk). Scale bars: A, B = 200μm, C, D = 50μm, E, F = 80 μm.

**Fig 3:**
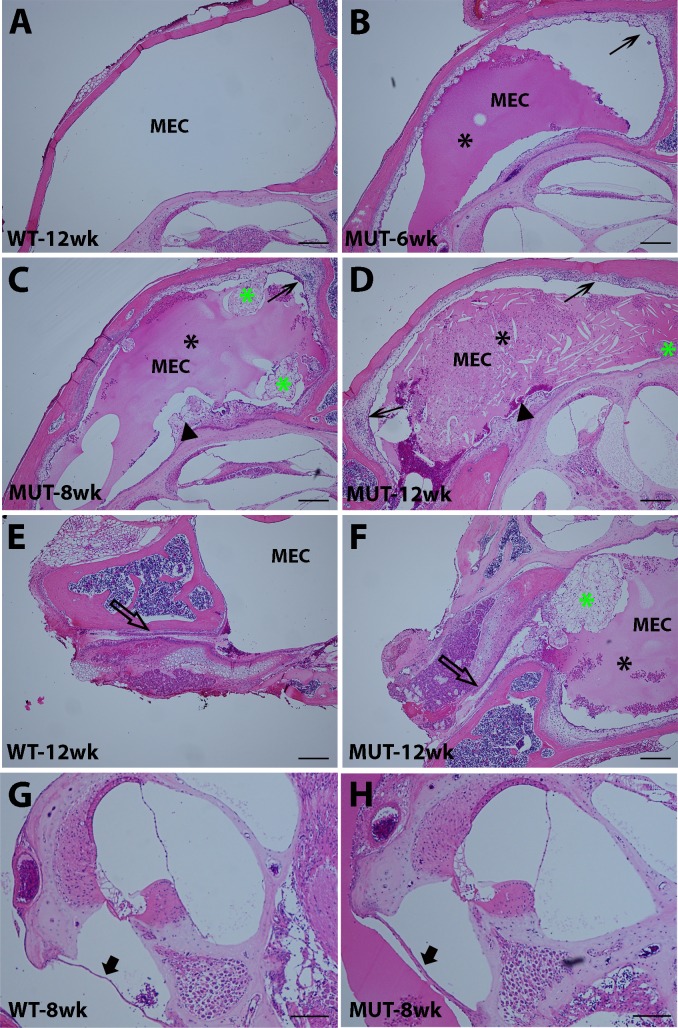
Development of otitis media in *Enpp1*^*asj/asj*^ mice. A-D: 6–12 week time course of middle ear pathology of *Enpp1*^*asj*^ mutant mice (B, C, D) compared with a 12-week-old control (A). At 6 weeks of age (B), an aqueous effusion (black asterisk) and a slightly thickened epithelium (arrow) are observed in the middle ear cavity of the *Enpp1*^*asj*^ mutant mice. At 8 weeks (C), the middle ear cavity of the *Enpp1*^*asj*^ mutant mouse is filled with an aqueous effusion (black asterisk) and contains amorphous tissue masses (green asterisk). The middle ear epithelium is much thickened (arrow), and the otic capsule exhibits regions of ectopic mineralization (arrowhead) with adjacent fibroblastic proliferation. At 12 weeks (D), the middle ear cavity of mutant mice is filled with pus-like secretions (black asterisk), a thickened epithelium (arrows), and an amorphous tissue mass (green asterisk). E, F: Eustachian tube morphology of mutant (F) and control (E) mice at 12 weeks of age. In mutant mice, an amorphous tissue mass (green asterisk) is present near the orifice of the Eustachian tube (empty arrow), which may impede Eustachian tube function and lead to an accumulation of effusion in the middle ear cavity. G, H: Cochlear morphology and round window membranes of mutant (H) and control (G) mice. Cochlear morphology is grossly normal in *Enpp1*^*asj*^ mutant mice compared with controls; however, the round window membrane (black arrow) of mutant mice is thicker. MEC: middle ear cavity. Scale bars: A, B, C, D, E, F = 200μm, G, H = 100μm.

All ears from *Enpp1*^*asj/asj*^ mice that had elevated ABR thresholds (n = 11) exhibited defects in the middle ear including retracted tympanic membranes and middle ear cavities filled with effusion (Figs 2 and 3, Table 3) and a thickened epithelium with fibrous polyps (Figs 2B, 2D and 3C, Table 3). In addition, older *Enpp1*^*asj/asj*^ mice exhibited excessive accumulation of discharge in the external ear canal (Fig 2H). All of the ears from the control *Enpp1*^*+/asj*^ and *Enpp1*^*+/+*^ mice examined (n = 12) showed a transparent tympanic membrane, a clear middle ear cavity lined with a thin epithelium.

**Table 3:** Histological assessment of the middle ears of the *Enpp1* mutant and control mice. MEE: Middle Ear Effusion; EH: Epithelial Hyperplasia; IC: Inflammatory Cells; ETO: Eustahian Tube Opening; EM: Ectopic Mineralization; CGC: Cluster of Goblet Cells.

Mouse ID	Age	Genotype	MEE	EH	IC	ETO	EM	CGC
4012L	1wk	Control	-	+	-	N/A	-	-
4017	1wk	Control	-	-	-	N/A	-	-
4015L	1wk	Control	-	-	-	N/A	-	-
4016L	1wk	Mutant	-	-	-	N/A	-	-
4016R	1wk	Mutant	-	-	-	N/A	-	-
4018L	1wk	Mutant	-	+	-	N/A	-	+
4022L	2wk	Control	-	-	-	N/A	-	-
4022R	2wk	Control	-	-	-	N/A	-	-
4019R	2wk	Control	-	-	-	N/A	-	-
4023L	2wk	Mutant	-	+	-	N/A	-	-
4023R	2wk	Mutant	-	+	-	N/A	-	-
4020L	2wk	Mutant	-	++	+	N/A	-	-
4008L	2wk	Mutant	-	+	-	N/A	-	-
4008R	2wk	Mutant	-	+	-	N/A	-	-
4007L	2wk	Mutant	-	+	-	N/A	-	+
4007R	2wk	Mutant	-	+	-	N/A	-	+
4002L	3wk	Control	-	-	-	N/A	-	-
4002R	3wk	Control	-	-	-	N/A	-	-
4001L	3wk	Control	-	-	-	N/A	-	+
4001R	3wk	Control	-	-	-	N/A	-	+
4004L	3wk	Mutant	-	-	-	N/A	-	-
4004R	3wk	Mutant	-	-	-	N/A	-	-
4000L	3wk	Mutant	-	-	-	N/A	-	-
4000R	3wk	Mutant	-	-	-	N/A	-	-
5055R*	3wk	Mutant	-	+	-	N/A	-	-
5055L*	3wk	Mutant	-	+	-	N/A	-	-
4193R*	6wk	Control	-	+	-	N/A	-	+
4195R*	6wk	Mutant	+	-	-	N/A	+	+
4094R*	6wk	Mutant	+++	+++	+	N/A	+	+++
3892L*	8wk	Control	-	-	-	-	-	
3893R*	8wk	Mutant	+++	+++	+	+++	+	++
3891R*	8wk	Mutant	+	+	-	N/A	+	-
3900R*	12wk	Control	-	-	-	N/A	+	-
3905L*	12wk	Mutant	+++	+++	++	N/A	+	+++
3905R*	12wk	Mutant	+	+	+	+	+	-
3899L*	12wk	Mutant	+	+++	++	+++	+	+++
3899R*	12wk	Mutant	++	+++	++	N/A	+	++
3883L*	26wk	Control	-	-	-	N/A	-	-
3883R*	26wk	Control	-	-	-	-	-	-
3887L*	26wk	Control	-	-	-	N/A	-	+
3887R*	26wk	Control	-	-	-	-	-	-
3884L*	26wk	Mutant	+++	++	++	++	-	+++
3884R*	26wk	Mutant	+	++	+	++	+	+
3885L*	26wk	Mutant	-	-	+	-	-	+
3885R*	26wk	Mutant	++	++	+++	++	-	++
2094R	30wk	Mutant	++	++	++	+	+	++
2096R	30wk	Mutant	+++	+++	+++	++	+	++
2098R	30wk	Mutant	+++	+++	++	+++	+	++

* Indicate ears with ABR data.

#### b. Increased goblet cell density and mucin secretion in *Enpp1*^*asj/asj*^ mice

**Fig 4:**
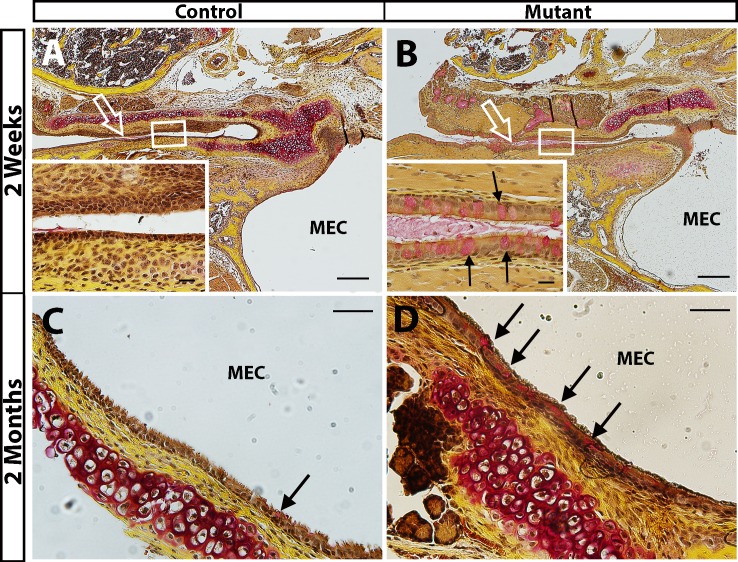
Increased density of goblet cells in *Enpp1*^*asj/asj*^ mice. Mayer's mucincarmine method was used to visualize goblet cells (stained red) in the epithelia lining the Eustachian tube (A, B) and middle ear cavity (C, D) of mutant and control mice. A, B: Few goblet cells are seen in the epithelia lining the Eustachian tube of littermate control mice (A, empty arrow points to Eustachian tube, insert shows higher magnification of the Eustachian tube epithelia). By contrast, goblet cells are present at high density in the epithelium lining the Eustachian tube of *Enpp1*^*asj*^ mutant mice (B, empty arrow points to Eustachian tube, magnified inset shows goblet cells, marked by arrows, in the Eustachian tube epithelia). C, D: More goblet cells are present in the epithelia in the middle ear cavity (MEC) of the *asj* mutant mouse (arrows in D) than in the control (C). Scale bars: A, B = 200 μm, C, D = 50 μm, A and B inserts = 20 μm.

In the epithelium lining the middle ear cavity and the Eustachian tube, mucins secreted by goblet cells build the first line of defense in protecting the host from invading pathogens; movements of the cilia then help to get rid of the pathogens, with mucins serving as a lubricant [24]. However, when chronic inflammation is present, some of the epithelial cells transdifferentiate to mucin-secreting goblet cells [24]. These additional goblet cells secrete excessive mucins, which accumulate in the middle ear cavity and lead to conductive hearing loss (otitis media). We used Mayer’s mucicarmine staining to detect the density of goblet cells in the *asj* mutant mice. Already at two weeks of age, an excess of goblet cells was observed around the opening of the Eustachian tube and in the Eustachian tube duct of mutant mice (Fig 4B). In the control mice, scattered goblet cells were seen around the opening of the Eustachian tube, but very few were detected along the Eustachian tube duct (Fig 4A). Similar observations were made in two-month-old adult mice: more goblet cells were present in the epithelia lining the middle ear cavity and the Eustachian tube in mutants (Fig 4D) than in controls (Fig 4C).

#### c. Impaired Eustachian tube function due to epithelia proliferation

Out of 10 ear preparations (8–30 weeks) that allowed us to observe the opening of the Eustachian tube in the middle ear cavity, 7 had amorphous tissues that could potentially block the Eustachian tube (Table 3) thereby disrupting middle ear pressure regulation and ciliary clearance of secretions.

#### d. Impaired middle ear epithelial clearance function due to excess mucin and loss of microciliary function

**Fig 5:**
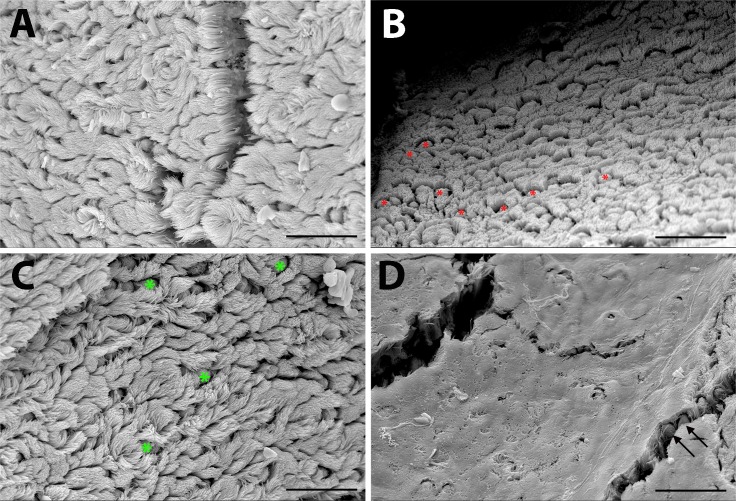
Scanning electron micrographs of epithelium lining the middle ear cavity. Compared with littermate controls (A), the middle ear epithelium in 1-month-old *Enpp1*^*asj*^ mutant mice (B) shows an increased number of goblet cells (red asterisks). Scattered goblet cells (green asterisks) and cilia are seen in the mucociliary epithelia of control mice at 6-months of age (C); however, a layer of mucin obscures most cilia and goblet cells (arrows) in age-matched *Enpp1*^*asj*^ mutant mice (D). Scale bars = 25μm.

Using scanning electron microscopy (Fig 5), we assessed the integrity of the mucociliary epithelium in 1-month-old and 6-month-old wild-type and *Enpp1*^*asj/asj*^ mice (n = 3 each genotype). The epithelium of one-month-old mutant mice had scattered areas with high densities of goblet cells (Fig 5B), which was confirmed by Mayer’s mucincarmine staining. Goblet cell density in 6-month-old mutants could not be determined because of the obscuring layer of mucin (Fig 5D). These results indicate that excessive mucin secreted by increased numbers of goblet cells and the hindering effect of the mucin layer on ciliary function in the *asj* mutant mice are contributing factors to the occurrence of otitis media.

### Over-calcification of middle ear structures (tympanosclerosis)

**Fig 6:**
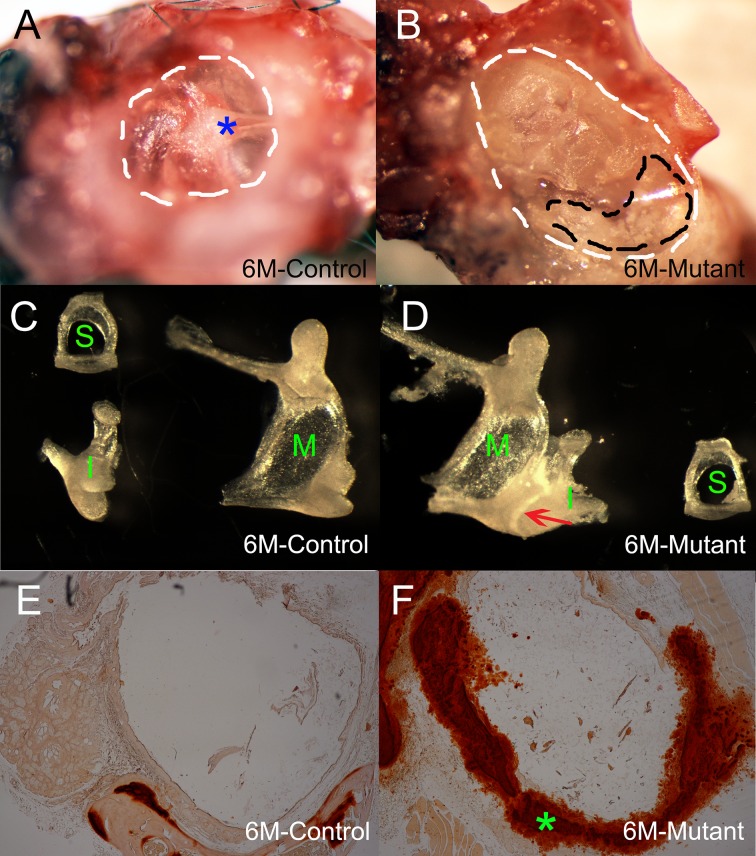
Calcification of middle ear structures (or tympanosclerosis) in *Enpp1*^*asj/asj*^ mice. In the control mice (A), the outer ear canal is clear without any discharge. The tympanic membrane appears to be transparent and malleus (blue asterisk) is clearly visible. In the age matched mutant mice (B), surrounding bone must be removed to expose the tympanic membrane, most of which is completely covered by discharge. White patches (inside black dashed lines) were observed on the tympanic membrane of the mutant mice (B). The ossicle bones of the age matched control and mutant mice appear to have similar morphology (C-D), but malleus and incus are fused in the mutant mice (D, red arrow). M: Malleus; I: Incus; S: Stapes. Alizarin red staining reveals extensive mineralization in the stapedial artery wall (green asterisk) in *Enpp1*^*as/asjj*^ mice (F), but not in the control mice (E).

6-month old control mice have transparent tympanic membrane (Fig 6A), mutant mice at the same age have retracted tympanic membrane due to the pressure of excessive discharge and white patch were observed at pars tensa of tympanic membrane in the mutant mice (Fig 6B). We dissected middle ear ossicles of 6-month-old *Enpp1*^*asj/asj*^ mice with littermate controls. Control mice have normal morphology of ossicles (Fig 6C). We found that stapes in the mutant mice have normal morphology and are freely removable from the round window (Fig 6D). Although malleus an incus has relative normal morphology, these two bones are fused (Fig 6D). The wall of the stapedial artery, which passes through the ring of the stapes, is thicker in *Enpp1*^*asj*^ mutant mice (Fig 2F) than controls (Fig 2E). Alarin red staining indicates excessive calcium deposition in the artery wall of the mutant mice (Fig 6F). Therefore, enlarged and stiffened stapedial artery could potentially impede the movement of the stapes and lead to impaired sound transmission to the inner ear. We observed overossification of round window ridge in the mutant mice (Fig 2B, Table 3), which may indirectly contribute to otitis media and hearing loss. We observed that the round window membrane in *Enpp1*^*asj*^ mutant mice (Fig 3H) was thicker than in control mice (Fig 3G).

### Bacterial infection does not play a role in the development of otitis media in *Enpp1*^*asj/asj*^ mice

The effusion was confined within the middle ear cavity and did not appear to extend through the round window into the inner ear. To detect if otitis media in the *asj* mutant mice was caused by bacterial infection, we performed gram staining in the freshly prepared middle ear sections. We failed to detect any pathogens from the middle ear cavities of the *asj* mutant mice (data not shown), suggesting a non-infection origin of the otitis media in the *asj* mutant mice.

## Discussion

### Otitis media-related ear pathology of *Enpp1*^*asj*^ mutant mice

Beginning at 6 weeks of age, an effusion starts to appear in the middle ears of *asj* mutant mice, and moderate epithelial proliferation is observed lining the middle ear cavity. The degree of middle ear effusion and epithelial thickening in *asj* mutant mice correlates with ABR thresholds. At 12 weeks of age the content of the effusion changes from serous to suppurative, with a corresponding increase in ABR thresholds. These effusions in the middle ear cavity may interfere with the normal vibration of the tympanic membrane and movement of the ossicle chain; with suppurative effusion having a much stronger effect. We observed discharge in the outer ear cavity starting around 4 months of age. All of the ears examined from seven 6~7-month-old *asj* mutant mice showed complete blockage of the outer ear canal by discharge, which could explain the secondary increase in ABR thresholds that occurs in *asj* mutant mice between 12 and 18 weeks of age.

Mucins are secreted by goblet cells that lay scattered in the epithelium of digestive, respiratory, urinary, and reproductive tracts either at a basal level or at a high level upon stimulation [25]. Mucins, together with the cilia that line the epithelia, protect the host by cleaning invading pathogens. A similar mechanism is applied by the middle ear mucociliary system to clear middle ear effusions [24]. Disturbed phosphate homeostasis can cause systematic inflammation. Inflammation within the middle ear cavity triggers secretion of mucin into the middle ear cavity and transdifferentiation of more epithelial cells into goblet cells, which lead to excessive effusion accumulation in the middle ear cavity. Continued chronic middle ear inflammation leads to tissue destruction and fibrosis. Thickened middle ear epithelia or fibrosis of epithelia, especially the epithelia around the opening of the Eustachian tube in the middle ear cavity can block the Eustachian tube and facilitate effusion accumulation. Although we don’t have supporting evidence, excessive discharge in the outer ear canal might be caused by inflammation in the external ear. Defective action of the cilia lining the middle ear epithelia is often associated with development of otitis media, as seen in patients with primary ciliary dyskinesia (PCD) [26]. Impaired mucociliary function and increased number of goblet cells in the middle ear cavity of the *asj* mutant mice, confirmed by SEM and Mayer’s mucicarmine staining, indicate that the middle ears of the *asj* mutant mice cannot maintain their ability to clear effusion, which therefore leads to conductive hearing loss. Overall, middle ear inflammation with effusion, amorphous tissue mass in the middle ear cavity, excessive discharge in the outer ear canal, and ectopic mineralization contribute to the conductive hearing loss in the *asj* mutant mice.

### Mineralization disorders and conductive hearing loss in *Enpp1*^*asj/asj*^ mice

Mineralization disorders due to abnormal phosphate levels have been associated with inflammation in several different diseases, including chronic kidney disease [27,28] and caridiovascular disease [29]. *Enpp1*^*asj*^ adds to a growing list of mouse mutations causing phosphate homeostasis disorders that have associated conductive hearing loss, including mutations of the *Ank*, *Phex*, *Rpl38*, and *Fgf23* genes. *Ank* (progressive ankylosis) encodes a multiple-pass transmembrane protein that regulates pyrophosphate levels, and *Ank* mutant mice were reported to have middle ear ossicle fusions and associated conductive hearing loss [30]. *ANK* mutations in human patients also have been reported with associated with conductive hearing loss [31]. PHEX (X-linked phosphate regulating endopeptidase) is an enzyme that is involved in regulating the balance of phosphate in the body. *PHEX* mutations are associated with X-linked hypophosphatemic rickets in human patients, with hearing loss as one of the symptoms [32–34]. Mice with *Phex* mutations exhibit hypophospatemia-related abnormalities and hearing impairment [35], which recently was shown to be associated with middle ear effusion and ciliary defects [8]. *Rpl38* (ribosomal protein L38) is not known to be directly involved in phosphate regulation; however, elevated organic phosphate levels in *Rpl38*^*Ts*^ mutant mice [4] suggest a potential but unknown function of *Rpl38* in phosphate homeostasis. *Rpl38*^*Ts*^ mutant mice have conductive hearing loss caused by ectopic ossification and cholesterol crystal deposition in the middle ear cavity, enlarged Eustachian tube, and chronic inflammation with effusion [4]. FGF23 (fibroblast growth factor 23) is a circulating hormone that controls phosphate and calcium homeostasis and bone mineralization. Abnormal serum levels of FGF23 lead to systemic pathologies in humans, including renal phosphate wasting diseases and hyperphosphatemia. FGF23-deficient mice show a mixed hearing loss and middle ear malformations [36], and changes in circulating FGF23 have been observed in humans and mice with ENPP1 and PHEX deficiencies [16,37].

ENPP1 produces inorganic pyrophosphate (PPi), an inhibitor of mineralization. Because PPi levels are markedly reduced in *Enpp1*^*asj/asj*^ mice [13], it is not surprising to see ectopic mineralization and calcification of middle ear tissues, as was observed in other soft tissues of these mutant mice [13]. Calcification of soft tissues within the middle ear cavity could contribute in various ways to the conductive hearing loss of *Enpp1*^*asj/asj*^ mice. The stapedial artery is an embryonic artery that disappears at the early embryo stage in humans but is conserved in mice through adult ages [38]. We observed a thickened stapedial artery wall in *Enpp1*^*asj*^ mutant mice, which is likely due to calcification as has been observed in other soft tissues. The thicker arterial wall is not likely to affect transport function, but because the artery passes through the ring of the stapes, its thickened wall could potentially impede the vibration of the stapes and contribute to the conductive hearing loss of mutant mice.

Abnormal mineralization can also cause otosclerosis and tympanosclerosis, two conditions that commonly lead to hearing loss in human patients. Disruption of bone homeostasis of the otic capsule can lead to otosclerosis [39], and the most common feature of otosclerosis is stapes fixation. Although we observed ectopic mineralization and bone deposition in the otic capsule of *asj* mutant mice starting from around 6 weeks of age, stapes fixation is absent from *Enpp1* mutant mice. Instead, we observed white patch on the tympanic membrane, malleus and incus fusion, which are typical symptoms of tympanosclerosis. We observed a thickened round window membrane in *Enpp1*^*asj/asj*^ mice at 8 weeks of age, which may increase the rigidity of the membrane and impede proper cochlear fluid movement and hair cell stimulation [40]. Therefore, *Enpp1*^*asj*^ mice can serve as a model for studying tympanosclerosis.

Decreased PPi levels in *Enpp1*^*asj/asj*^ mice also lead to otitis media, perhaps the most important factor contributing to the hearing loss, but the underlying mechanism of pathology is uncertain. ENPP1 deficiency is known to cause elevated serum levels of FGF23 in *Enpp1* mutant mice [16], and excess FGF23 secreted in the middle ear may trigger mucoperiosteum proliferation, which may contribute to the development of otitis media. In support of this possibility, mucoperiosteum proliferation in *Enpp1*^*asj/asj*^ mice is remarkably enhanced around the regions of the otic capsule that exhibit ectopic mineralization. *Enpp1*^*asj*^ mice provide a tool to unravel the underlying mechanism of the development of otitis media that is associated with abnormal phosphate homeostasis.

This is the first report of hearing loss and ear pathology associated with a mutation of the mouse *Enpp1* gene. The conductive hearing loss of *Enpp1*^*asj*^ mutant mice provides a new animal model for studying otitis media and tympanosclerosis related to mineralization defects. It also provides a specific model for understanding the hearing loss recently reported to be a clinical feature associated with human *ENPP1* mutations [17,18].
